# Secondary attack rate following on-site isolation of patients with suspected COVID-19 in multiple-bed rooms

**DOI:** 10.1186/s13756-024-01430-4

**Published:** 2024-07-06

**Authors:** Silvio Ragozzino, Richard Kuehl, Karoline Leuzinger, Pascal Schläpfer, Pascal Urwyler, Ana Durovic, Sandra Zingg, Matthias von Rotz, Manuel Battegay, Andreas F Widmer, Hans H Hirsch, Stefano Bassetti, Sarah Tschudin-Sutter

**Affiliations:** 1 Division of Infectious Diseases and Hospital Epidemiology, University Hospital Basel, University Basel, Basel, Switzerland; 2 Clinical Virology, Laboratory Medicine, University Hospital Basel, University of Basel, Basel, Switzerland; 3 Transplantation and Clinical Virology, Department Biomedicine, University of Basel, Basel, Switzerland; 4 Division of Internal Medicine, University Hospital Basel, University Basel, Basel, Switzerland; 5 Division of Infectious Diseases and Hospital Epidemiology, University Hospital Basel, Petersgraben 4, Basel, CH-4031 Switzerland

**Keywords:** SARS-CoV-2, COVID-19, Isolation on site, Droplet and contact precautions, Shared rooms, Secondary attack rate, Disease outbreaks, Healthcare-associated COVID-19, Whole-genome sequencing, Infection prevention and control

## Abstract

The implementation of isolation precautions for patients with suspected Coronavirus Disease 2019 (COVID-19) and pending test results is resource intensive. Due to the limited availability of single-bed rooms at our institution, we isolated patients with suspected COVID-19 together with patients without suspected COVID-19 on-site in multiple-bed rooms until SARS-CoV-2-test results were available. We evaluated the likelihood of SARS-CoV-2 transmission to individuals sharing the room with patients isolated on-site. This observational study was performed at the University Hospital Basel, Switzerland, from 03/20 − 11/20. Secondary attack rates were compared between patients hospitalized in multiple-bed rooms and exposed to individuals subjected to on-site isolation precautions (on-site isolation group), and patients exposed to individuals initially not identified as having COVID-19, and not placed under isolation precautions until the diagnosis was suspected (control group). Transmission events were confirmed by whole-genome sequencing. Among 1,218 patients with suspected COVID-19, 67 (5.5%) tested positive for SARS-CoV-2. Of these, 21 were isolated on-site potentially exposing 27 patients sharing the same room. Median contact time was 12 h (interquartile range 7–18 h). SARS-CoV-2 transmission was identified in none of the patients in the on-site isolation group vs. 10/63 (15.9%) in the control group (*p* = 0.03). Isolation on-site of suspected COVID-19-patients in multiple-bed rooms avoided single-room occupancy and subsequent in-hospital relocation for many patients without confirmed SARS-CoV-2-infection. The absence of secondary transmission among the exposed patients in the on-site isolation group allows for assessment of the risk/benefit ratio of this strategy given the limitation of a small sample size.

## Introduction

During the Coronavirus Disease 2019 (COVID-19) pandemic, a substantial influx of patients with suspected COVID-19 placed significant strain on many healthcare systems, leading to a saturation of hospital resources [1, 2]. The limited availability of single-bed rooms in numerous settings presents additional challenges in implementing isolation precautions, particularly for patients with suspected COVID-19, during the interval while awaiting test results [3]. In response to these limitations, our institution adopted a strategy of on-site isolation for patients with suspected COVID-19 in multiple-bed rooms alongside patients without suspected COVID-19 until results of SARS-CoV-2 molecular assays became available. This study aims to evaluate the feasibility of this approach by assessing the secondary attack rate among patients who shared multiple-bed rooms with an individual initially suspected and subsequently confirmed to have COVID-19. As comparator, we examined a group of patients exposed to individuals initially not identified as having COVID-19, and consequently not placed under isolation precautions until the diagnosis was suspected or established. Whole-genome sequencing (WGS) was performed to confirm transmission events following exposure in the same multiple-bed room. We further sought to identify risk factors related to transmission.

## Methods

### Study design and setting

This single-center retrospective cohort study was conducted at the University Hospital Basel, Switzerland, during the first two waves of the COVID-19 pandemic, between March and November 2020. The University Hospital Basel is a 750-bed tertiary academic care center, admitting more than 40,000 adult patients annually. It was one of the main healthcare centers providing care for COVID-19 patients during the pandemic in the canton of Basel-Stadt with a population of approximately 250,000 residents. Due to the limited availability of single-bed rooms, newly admitted patients with suspected COVID-19 were isolated on-site in multiple-bed rooms until SARS-CoV-2-test results from nasopharyngeal swabs were available. As a quality assessment project, the Ethics Commission of Northwestern and Central Switzerland (EKNZ) confirmed that no approval was required (EKNZ-Request-2021-00550).

### Study population, isolation strategy and definitions

During the study period, universal SARS-CoV-2 screening was performed from April 1st to June 14th 2020. It was then stopped due to the low number of detected SARS-CoV-2 infections among asymptomatic patients (i.e. 0.1%) [4]. Thereafter, SARS-CoV-2 testing was driven by symptoms with a very low threshold for clinical suspicion being promoted at our institution. The suspicion of COVID-19 was based on the presence of compatible clinical symptoms, such as fever and/or respiratory symptoms and/or other unexplained symptoms. The on-site isolation group consisted of patients newly admitted with suspected COVID-19, subjected to on-site isolation precautions until infection was confirmed. After confirmation of SARS-CoV-2-infection, patients were reallocated to the COVID-19 cohort ward. Contacts were defined as patients sharing the same multiple-bed room and, therefore, being exposed to one of these index-patients for more than 15 min. On-site isolation procedures encompassed a combination of droplet and contact precautions. The patient area was delineated by the use of room dividers or floor markings, and dedicated toilets were assigned. Isolation measures included the use of surgical masks, gloves, and gowns for all individuals directly interacting with the patient or coming into contact with their immediate surroundings. Patients were required to wear surgical masks. On-site isolation was not applied for patients not compliant with the use of surgical masks. Non-immunocompromised patients without suspected COVID-19 were accommodated in the same room.

On the other hand, the control group consisted of patients initially not identified as having COVID-19, and consequently not placed under isolation precautions until the diagnosis was suspected or established. Following the diagnosis of the index patient, their contacts, as per the aforementioned definition, were placed under preemptive droplet precaution measures in single-rooms and were systematically screened for SARS-CoV-2 infection. Screening of contact patients at the time of the study consisted of SARS-CoV-2 molecular testing at day 0, 3 and 7. Number of air exchanges at our center is 6 per hour in high-risk wards, such as the intensive care unit and 1–4 per hour in conventional wards.

### Data collection

Pertinent clinical and microbiological data of patients included in the on-site isolation and control group (index patients and their contacts) were collected. The medical records of all contact patients were screened for subsequent diagnosis of SARS-CoV-2-infection during the course of hospitalization or on re-admission to our institution.

### Outcomes

The primary outcome was the secondary attack rate, defined as the proportion of contact patients diagnosed with SARS-CoV-2 infection after having been exposed to an index patient hospitalized in the same multiple-bed room. Secondary attack rates were compared between on-site isolation group and control group. The secondary outcome were risk factors for transmission events after exposure.

### SARS-CoV-2 nucleic acid testing and whole genome sequencing

SARS-CoV-2 quantitative nucleic acid testing (QNAT) was done as described previously [5, 6]. To examine potential transmission events between index patients and contact patients hospitalized in the same multiple-bed room, WGS of SARS-CoV-2 was carried out following the methods described previously [7]. Strain identity was defined as no more than one single-nucleotide polymorphism difference between strains by phylogenetic analyses [8].

### Statistical analysis

To assess the comparability between the two groups (i.e. on-site isolation group and control group), baseline characteristics were compared between index and contact patients belonging to each group. Further, potential risk factors for transmission were assessed by comparing contact patients with and without transmission events after exposure. Continuous variables were expressed as median and interquartile range (IQR) and categorical variables as frequencies and percentages. Differences between on-site isolation and control group were investigated with the χ2 and Fisher exact test (where appropriate) for categorical variables and the Mann-Whitney U test for continuous variables. Two-sided *P* values of less than 0.05 were considered as statistically significant. SPSS version 20 (IBM Corporation) was used for statistical analysis.

## Results

**Fig. 1:**
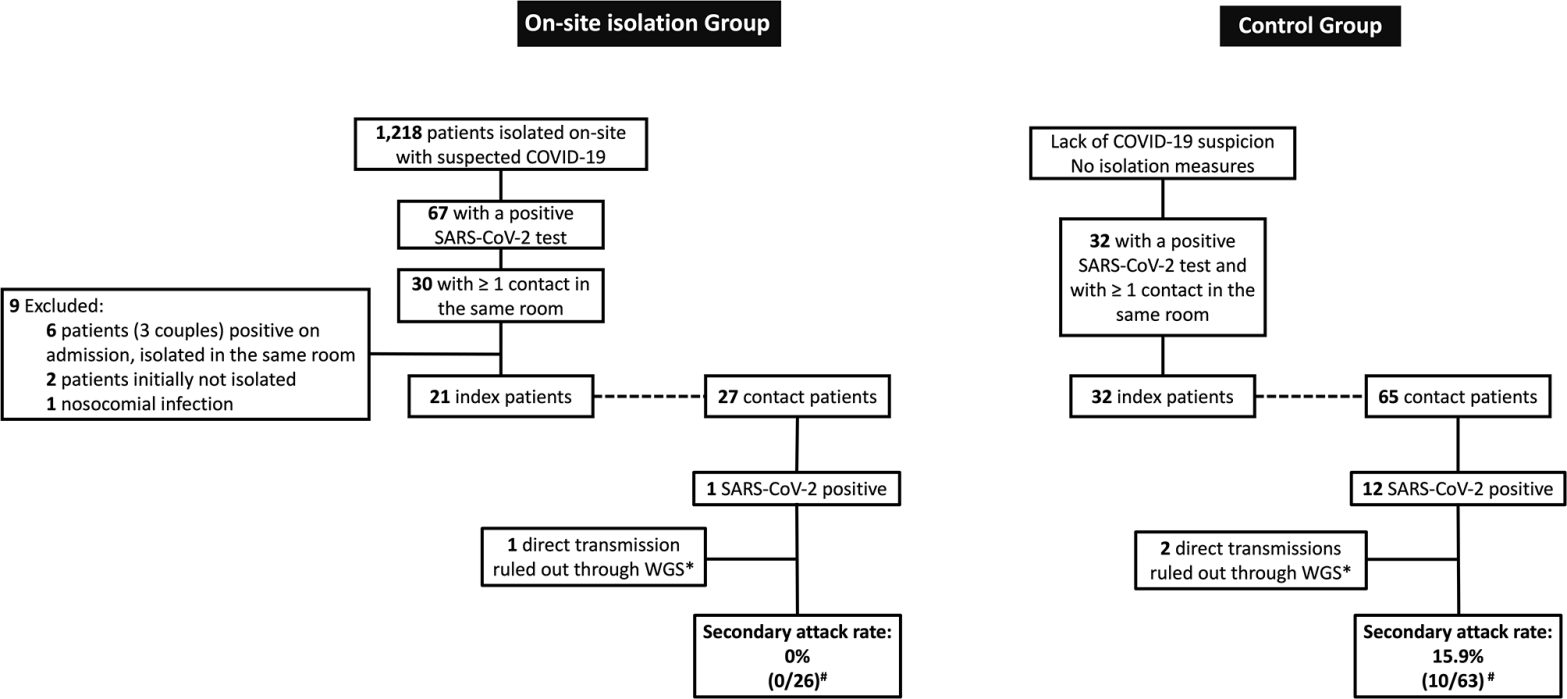
Flow-chart of the patients included in the study. * WGS available for seven index-contact pairs: three direct transmissions ruled out, four confirmed. For the remaining six events, no sequencing results were available. ^#^ Patients where direct transmission was ruled out by WGS were excluded from numerator and denominator counts. WGS: whole-genome sequencing

Between March and November 2020, 1,218 patients were admitted to the University Hospital Basel with suspected COVID-19 and were isolated on-site until SARS-CoV-2 QNAT-test results were available. Among them, 67 patients (5.5%) were confirmed to be SARS-CoV-2 positive. Twenty-one of these index patients shared the room with one or more contacts before conventional COVID-19-isolation precautions were put in place, potentially exposing 27 patients to SARS-CoV-2 transmission (on-site isolation group). The control group consisted of 32 COVID-19 patients, initially without any isolation precautions due to lack of clinical suspicion, who exposed 65 contact patients. The flow-chart of the included patients is shown in Fig. 1. Overall, we identified 13 possible transmission events, one in the on-site isolation group (1/27; 3.7%) and 12 in the control group (12/65; 18.5%). Sequencing analyses were available for seven index-contact patient pairs (53.8%). In three cases (one in the on-site isolation group and two in the control group) a direct transmission event could be ruled out. Therefore, no confirmed SARS-CoV-2 transmission occurred in the on-site isolation group, as compared with a secondary attack rate of 15.9% (10/63) in the control group (*p* = 0.03). Median contact time differed between the on-site isolation group and the control group (11.5 h, IQR 6.5–17.7 h vs. 20.4 h, IQR 12.4–42.0, *p* < 0.001). A comparison of the clinical characteristics of index and contact patients between the two study groups is shown in Table 1. Age and sex distribution were similar among both index and contact patients across the cohorts, with a uniform predominance of male sex in all groups. Index patients belonging to the control group had a higher frequency of chronic cardiovascular diseases, higher viral loads, and longer hospital stay until SARS-CoV-2 QNAT positivity, as compared with the index patients belonging to the on-site isolation group. Index patients isolated on-site more often presented with dyspnea or need for oxygen supplementation. SARS-CoV-2 testing was performed in nine out of 27 patients (33.3%) in the on-site isolation cohort vs. 41/65 (63.1%) in the control group, *p* = 0.01.

**Table 1:** Comparison of the main clinical and virological features of index and contact patients between the on-site isolation and control group

	Index patients		*P*-value^#^	Contact patients		*P*-value^#^
	On-site isolation group (*n*=21)	Control group (*n*=32)		On-site isolation group (*n*=27)	Control group (*n*=65)	
Age (years), median (IQR)	73.0 (63.5-77.0)	73.5 (63.0-79.5)	0.41	71 (57.0-78.0)	70 (60.5-76)	0.61
Male, n (%)	17 (81.0)	19 (59.4)	0.10	21 (77.8)	40 (61.5)	0.13
Comorbidities, n (%)						
Chronic lung disease	4 (19.0)	5 (15.6)	0.95	4 (14.8)	9 (13.8)	0.79
Diabetes	6 (28.6)	9 (28.1)	0.88	8 (29.6)	12 (18.5)	0.42
Hypertension	16 (76.2)	23 (71.9)	0.35	15 (55.6)	38 (58.5)	0.52
Chronic cardiovascular disease	5 (23.8)	16 (50.0)	0.03	13 (48.1)	21 (32.3)	0.25
Chronic renal disease	4 (19.0)	7 (21.9)	0.74	6 (22.2)	6 (9.2)	0.26
Chronic liver disease	2 (9.5)	1 (3.1)	0.56	3 (11.1)	5 (7.7)	0.69
Chronic neurological impairment/dementia	2 (9.5)	2 (6.3)	0.46	5 (18.5)	7 (10.8)	0.46
Cancer	2 (9.5)	6 (18.8)	0.44	5 (18.5)	13 (20.0)	0.72
HIV/ Immunosuppressive treatment	2 (9.5)	2 (6.3)	0.99	1 (3.7)	6 (9.2)	0.43
Length of stay* (days), median (IQR)	0 (0-1)	1 (0-5.75)	0.02	8 (4-11)	10 (4-20.5)	0.24
Ct-value, median (IQR)	30.5 (26.4 – 33.3)	22.8 (19.0-29.7)	0.005	-	-	-
Symptoms, n (%)						
Cough	11 (64.7)	11 (68.8)	0.81	-	-	-
Dyspnea	8 (47.1)	2 (12.5)	0.04	-	-	-
Fever	14 (82.4)	10 (62.5)	0.19	-	-	-
Oxygen supplementation	13 (61.9)	11 (34.4)	0.05	-	-	-
Death, n (%)	0 (0.0)	5 (15.6)	0.14	-	-	-
Ward, n (%)	-	-	-			0.01
Surgery				1 (3.7)	9 (13.8)	
Medicine				24 (88.9)	37 (56.9)	
ICU				2 (7.4)	19 (26.2)	
Four-bed room, n (%)	-	-	-	14 (51.9)	31 (47.7)	0.72
Adjacent bed to index, n (%)	-	-	-	17 (63.0)	42 (64.6)	0.88
Duration of contact (hours), median (IQR)	-	-	-	11.5 (6.8-17.7)	20.4 (12.4-42.0)	<0.001
SARS-CoV-2 test performed, n (%)	-	-	-	9 (33.3)	41 (63.1)	0.01
SARS-CoV-2 positive, n (%) After exclusion through WGS, n (%)	-	-	-	1 (3.7) 0 (0.0)	12 (18.5) 10 (15.9)^&^	0.10 0.03

* Length of stay until SARS-CoV-2-PCR-positivity for index cases. Global length of stay for contacts

^#^ χ2 and Fisher exact test for categorical variables and Mann-Whitney U test for continuous variables

^&^ Patients where direct transmission was ruled out by WGS were excluded from numerator and denominator counts (10/63, 15.9%)

IQR: interquartile range; HIV: Human Immunodeficiency Virus; Ct-value: cycle threshold value (number of cycles required for the fluorescent signal to cross the threshold in a real time PCR assay; Ct levels are inversely proportional to the amount of target nucleic acid in the sample, i.e. the lower the Ct level the greater the viral load); ICU: intensive care unit; WGS: whole-genome sequencing

A comparison between contact patients who developed SARS-CoV-2 infection and those who did not is detailed in Table 2. Underlying malignancy and exposure to significantly higher viral loads were more frequent among SARS-CoV-2-positive contact patients. No significant differences were observed regarding the duration of exposure. SARS-CoV-2 positive contact patients were more often placed in an adjacent bed to the index patient, however, without reaching statistical significance.

**Table 2:** Comparison between contact patients who subsequently developed SARS-CoV-2 infection and those who did not

Characteristics	Positive contacts (*n*=10)^#^	Negative contacts (*n*=79)	*P*-value*
Age (years), median (IQR)	73.5 (59.0-82.5)	70.0 (57.0-76.0)	0.21
Male, n (%)	6 (60.0)	52 (65.8)	0.73
On-site isolation, n (%)	0 (0.0)	26 (32.9)	0.03
Cancer, n (%)	6 (60.0)	11 (16.4)	0.006
Length of stay (days), median (IQR)	13.5 (12.0-21.0)	8.0 (3.0-15.0)	0.01
Ward, n (%)			0.88
Surgery	1 (10.0)	8 (10.1)	
Medicine	6 (60.0)	53 (67.1)	
ICU	3 (30.0)	18 (22.8)	
Four-bed room, n (%)	5 (50.0)	40 (50.6)	1.0
Adjacent bed to index, n (%)	8 (80.0)	48 (60.8)	0.31
Duration of contact (hours), median (IQR)	14.7 (6.3-66.3)	17.7 (9.0-26.8)	0.93
Ct-value index, median (IQR)	21.3 (15.4-23.1)	27.9 (23.4-32.5)	0.001

^#^ The three positive contacts, for which a direct transmission was ruled out through WGS, were not included in this table

* χ2 and Fisher exact test for categorical variables and Mann-Whitney U test for continuous variables

IQR: interquartile range; ICU: intensive care unit; Ct-value: cycle threshold value (number of cycles required for the fluorescent signal to cross the threshold in a real time PCR assay; Ct levels are inversely proportional to the amount of target nucleic acid in the sample, i.e. the lower the Ct level the greater the viral load)

## Discussion

On-site isolation of patients with suspected COVID-19 in multiple-bed rooms, applied over a restricted timeframe, was not associated with secondary transmission events in our setting and could represent an adequate alternative isolation strategy, especially during a shortage of single-bed rooms and/or lacking availability of rapid SARS-CoV-2 QNAT results.

A similar approach was previously implemented for other viral respiratory infections and revealed similar results [9, 10]: Birrer et al. evaluated the introduction of on-site droplet precautions in a tertiary-care center during the 2018/19 influenza season and did not find an increased rate of nosocomial infections as compared with the standard single-room isolation strategy [9]. To our knowledge this is the first study reporting data on the safety and feasibility of this strategy in the setting of suspected COVID-19. In line with the World Health Organization recommendations [11], we implemented a comprehensive testing approach, thus only a small proportion of patients with symptoms compatible with COVID-19 were confirmed to have a SARS-CoV-2 infection at our center. The on-site isolation strategy allowed us to avoid single-room occupancy and prevent the subsequent in-hospital relocation of a large number of patients without confirmed infection.

The secondary attack rate among the control group was similar to previous studies performed during the same period in hospital and community settings [12, 13]. According to our results, the main determinant of SARS-CoV-2 transmission seems to be the viral load of the index patients rather than the contact time or the proximity of the contact patients to the index in the same room. In this respect, the lower viral load observed among the index-patients in the on-site isolation cohort, could have significantly contributed to the success of this strategy. This difference in viral burden, however, is in accordance with the natural course of COVID-19: patients with SARS-CoV-2 infection usually develop an exacerbation of symptoms requiring medical attention in late stages of the disease when the viral load and thus transmissibility is reduced and immunological pathogenesis is predominant [14]. On the other hand, index-patients of the control group consisted mainly of nosocomial infections and/or pre- or paucisymptomatic phase, generally related with high viral burden [15, 16]. Longer contact times, previously identified as risk factor for SARS-CoV-2 transmission [13], seemed to play a less prominent role in our cohort. However, median contact time among the on-site isolated patients was significantly shorter as compared with the control group. Nowadays, the broad availability of rapid SARS-CoV-2 QNAT results would allow for even shorter exposure times, reinforcing the safety of this approach.

Interestingly, among the pairs of index-contact patients with sequencing results available, only 57% (4/7) of the transmission events could be supported by phylogenetic data. Similar results were found in an outbreak investigation carried out at our center, where approximately half of the transmission events were confirmed by WGS analysis, suggesting that the remaining cases most likely reflect community-acquired infections randomly detected by broad screening efforts [17].

This study has several limitations including the small sample size and the retrospective design. Formally, the clinical impact of the on-site isolation would have been better evaluated through the comparison with a control group of patients with suspected COVID-19 submitted to conventional pre-emptive isolation measures. Such a control group was not available given the COVID-19 surge during the study period and the early implementation of the on-site isolation strategy. The lack of transmission events recorded in our study, points to the challenges in terms of the required sample size to adequately power a well-designed interventional study. Estimates of the secondary attack rate among the control group may have been hampered by the presence of preexisting asymptomatic or presymptomatic infection. Therefore, transmission events were first postulated based on clinical plausibility (exposure and chronological onset of symptoms and/or test positivity) and then confirmed or ruled out through sequencing, if possible. Further, this study was performed during the circulation of the wild type and pre-alpha variants of SARS-CoV-2 and in the pre-vaccine era limiting its generalizability to other variants with potentially higher transmissibility [16, 18] and populations with higher levels of immunity. Nevertheless, a continuous risk assessment of this strategy at our center did not point to an increase of healthcare acquired SARS-CoV-2 infections. SARS-CoV-2 testing was only performed in a small proportion of the contact patients in the on-site isolation group. This may be due to several reasons, such as earlier hospital discharge or more difficult follow-up in case of room or ward transfer linked to the lack of proper labelling of these patients. This aspect could introduce a detection bias and, indeed, if missing data were excluded from the analysis, there would be no significant difference in the rates of confirmed secondary transmission between the two groups. However, the hospital’s testing site was the most important testing site within the city at that time, increasing the likelihood of detecting patients with symptomatic infections also in an outpatient setting. Sequencing results were not available for all potential transmission events and therefore the secondary attack rate in the control group could be overestimated. In favor of patient safety, we choose to err on the side of assuming transmission at our institution had occurred rather than not occurred. However, the only epidemiologically suspected transmission event in the intervention group could be ruled out, supporting this approach. We further acknowledge that the laboratory developed nucleic acid test used for SARS-CoV-2 quantification was not calibrated against an international reference standard [19].

## Conclusions

Isolation on-site of suspected COVID-19-patients in multiple-bed rooms could be a feasible and safe strategy. It avoids single-room occupancy and subsequent in-hospital relocation for many patients, ensuring a better quality of care and significant cost reductions for the healthcare system. Further specifically designed prospective studies in a larger clinical context and with the current circulating SARS-CoV-2 variants are needed to document the efficacy of this strategy. Thereafter, an additional intriguing question would be whether COVID-19 patients can be isolated on-site during the entire length of stay.

## Data Availability

The datasets used and analysed during this study are available from the corresponding author on reasonable request.

## Abbreviations

COVID-19: Coronavirus disease 2019
EKNZ: Ethics Commission of Northwestern and Central Switzerland
IQR: Interquartile range
QNAT: Quantitative nucleic acid testing
SARS-CoV-2: Severe acute respiratory syndrome coronavirus 2
WGS: Whole-genome sequencing
